# Congenital hypotrichosis caused by compound heterozygous variants in the LSS gene in a Chinese patient with strabismus: case report

**DOI:** 10.3389/fped.2025.1512646

**Published:** 2025-04-29

**Authors:** Linlin Bao, Qian Li, Zhicao Yue, Fang Yang

**Affiliations:** ^1^ Department of Dermatology, Shenzhen People’s Hospital (The Second Clinical Medical College, Jinan University; The First Affiliated Hospital, Southern University of Science and Technology), Shenzhen, Guangdong, China; ^2^ Candidate Branch of National Clinical Research Center for Skin Diseases, Department of Dermatology, Shenzhen People's Hospital, Shenzhen, Guangdong, China; ^3^ Prenatal Diagnosis Center, Department of Obstetrics and Gynaecology, Sixth Medical Center, Chinese PLA General Hospital, Beijing, China; ^4^ Department of Cell Biology and Medical Genetics, International Cancer Center, and Guangdong Key Laboratory for Genome Stability and Disease Prevention, Shenzhen University Medical School, Shenzhen, Guangdong, China

**Keywords:** genetic diagnosis, hypotrichosis, strabismus, LSS, genotype-phenotype

## Abstract

**Background:**

Lanosterol synthase (LSS) is essential for cholesterol biosynthesis and impacts embryonic development and growth. *LSS* gene variants have been associated with various conditions such as congenital hypotrichosis and cataracts, but the genotype-phenotype relationship remains not well understood.

**Case presentation:**

Herein, we report an 8-year-old boy presenting with congenital hypotrichosis and intermittent exotropia, but without any ocular movement abnormalities or cataracts. His hair exhibited sparse distribution with a yellow color, reduced strength, and minimal growth. Scanning electron microscopy revealed abnormal keratinization of the hair shafts, characterized by irregular, jagged scales and raised edges. Whole-exome sequencing identified compound heterozygous missense variants in the *LSS* gene: c.1303C>T (p.Arg435Cys) and c.386G>A (p.Arg129Gln). Three-dimensional protein modeling revealed that these variants affect highly conserved amino acid residues and are predicted by computational tools to destabilize the protein. Based on ACMG guidelines, both variants were classified as likely pathogenic, consistent with the patient's phenotype.

**Conclusion:**

We present a rare case of *LSS*-related hypotrichosis with strabismus and a novel c.386G>A variant has not been reported, which broadens the understanding of *LSS* gene variants and their phenotypic spectrum, enhancing insights into the genotype-phenotype relationship in *LSS*-related conditions.

## 1 Introduction

Cholesterol is essential for maintaining cell membrane integrity and hormone production. Its biosynthesis involves several enzymatic steps, with lanosterol synthase (LSS) playing a crucial role. In this process, LSS catalyzes the cyclization of (S)-2,3-oxidosqualene to form lanosterol, which serves as a key precursor in cholesterol biosynthesis. Lanosterol is subsequently converted to cholesterol through multiple steps, including demethylation, double bond reduction, and side chain modifications (1, 2). Variants in the *LSS* gene can disrupt this process, leading to cholesterol deficiency and accumulation of intermediates. *LSS* gene variants are linked to a variety of disorders, such as congenital cataracts, alopecia-intellectual disability syndrome, hypotrichosis simplex, and mutilating palmoplantar keratoderma, that have varying dermatological, ocular, and neurological symptoms (2–5).

Despite increased attention to the pathogenicity of *LSS* variants, precise genotype–phenotype correlations remain unclear (6–8). Compound heterozygous or homozygous *LSS* variants are associated with hypotrichosis and neurodevelopmental disorders, including intellectual disability. Recent reports also describe cases of *LSS*-related hypotrichosis with congenital cataracts (9–11). However, hypotrichosis with strabismus is rare, with only one case reported in 1991 (12). Herein we present the case of a Chinese boy with congenital hypotrichosis and strabismus. Whole-exome sequencing identified compound heterozygous variants (p.Arg435Cys and p.Arg129Gln) in the *LSS* gene, which correlate with the patient's phenotype and expand the understanding of *LSS* variants and their associated clinical manifestations.

## 2 Case presentation

### 2.1 Methods

#### 2.1.1 Patient

Data were collected from a boy diagnosed with hypotrichosis and strabismus at the Department of Dermatology, Shenzhen People's Hospital. The study adhered to the principles of the Declaration of Helsinki, and written informed consent was obtained from the patient's guardians prior to participation.

#### 2.1.2 Examination of hair with scanning electron microscopy (SEM)

Hair samples were collected from the patient, specifically from the posterior vertex, and cut into 1-cm segments, processed, and fixed prior to SEM analysis. SEM was performed using a high-resolution scanning electron microscope (Thermo Scientific APREO S, MA, USA) in the Department of Cell Biology and Medical Genetics, Shenzhen University Medical School.

#### 2.1.3 Assessment of strabismus

The ocular deviation was assessed using a TSJ-IV-A prism (Changchun Optical Instrument Co., China) under best-corrected refractive conditions. A sequential monocular prism placement method was applied after 30 min of left eye occlusion. The prism was placed before the deviating eye while the patient fixated on standardized near (33 cm) and distance (5 m) targets. An alternating cover-uncover test was performed, gradually increasing prism power from 10 prism diopters (PD) in 2-PD increments until no movement was observed upon covering the sound eye. The final prism power required to neutralize exodeviation was recorded in PD. Both eyes exhibited symmetrical 18 PD base-in prism requirements for orthotropia at all fixation distances.

#### 2.1.4 Whole-exome sequencing analysis

Whole-exome sequencing analysis (MyGenostics Inc., China) was performed on the proband and his family members (parents and sister). Genomic DNA was extracted from 2 ml of peripheral blood using the RelaxGene Blood DNA System DP319-02 (Tiangen, China), following the manufacturer's instructions. A genomic library was constructed with a standard library construction kit, and whole-exome sequencing was carried out using target sequence capture probes (MyGenostics, GenCap, China) to cover all exons of all genes. Sequencing was conducted on the Illumina NextSeq 500 platform (Illumina Inc., USA), and approximately 2.4 gigabases of mappable data were generated with a mean coverage depth of 315.58X across the target region. Variants with minor allele frequencies of <0.05 in population databases (including 1000 Genomes, ESP6500, ExAC, GnomAD, and the MyGenostics in-house database) as well as those predicted to impact protein-coding or splicing were included in the analysis. Suspected candidate variants were evaluated based on the disease's genetic pattern and the patient's clinical presentation. Candidate genes were further validated through Sanger sequencing (13).

#### 2.1.5 Structural analysis of LSS variants

Three-dimensional protein modeling and analysis were conducted using online tools to evaluate the protein structure, identify conserved and functional domains, and perform multiple sequence alignments. A 3D model of human LSS (PDB: 1W6K) was generated using SWISS-MODEL (https://swissmodel.expasy.org/), and molecular graphics were created with PyMOL software. Additionally, bioinformatics platforms such as REVEL, PolyPhen-2, MutationTaster, and AlphaMissense were employed to predict and annotate functional variants in the protein.

### 2.2 Results

#### 2.2.1 Clinical presentation

**Figure 1:**
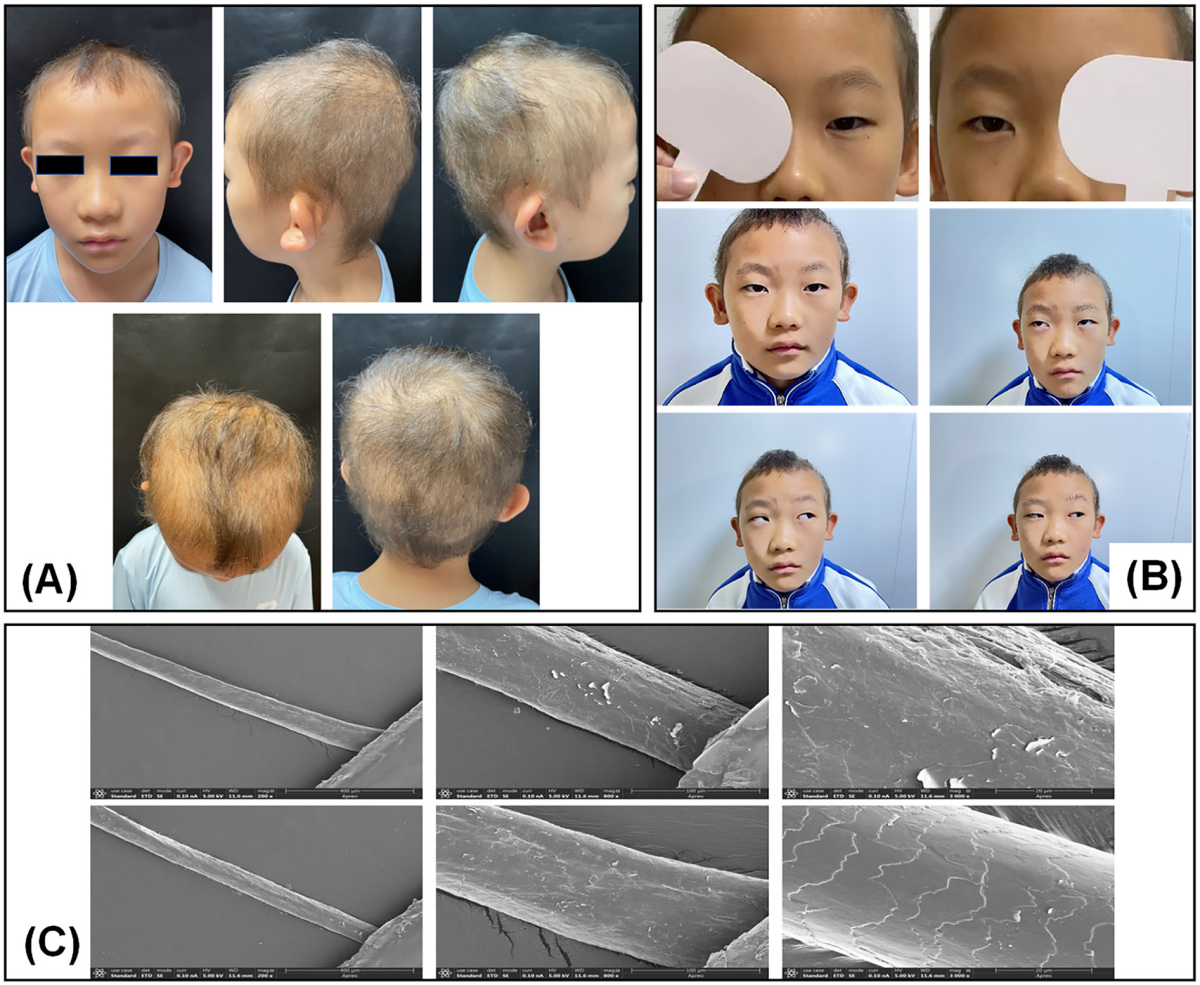
Clinical phenotypic features of the patient. **(A)** Clinical presentation. **(B)** Assessment of strabismus. **(C)** Scanning electron microscopy image of the patient's hair specimen.

The patient was an 8-year-old male who had experienced sparse scalp hair and hair loss since birth, indicative of congenital hypotrichosis. His hair was sparsely distributed with a yellow tint and exhibited reduced strength and minimal growth (Figure 1). However, his eyelashes, eyebrows, and body hair were normal, and his nails showed no abnormalities. Subsequent examinations at other hospitals revealed that the patient had intermittent exotropia (see Figure 1B and Supplementary Figure 1 for details). The cover-uncover test showed that both eyes returned to the orthotropic position from an exotropia position, with equal deviation angles of approximately −15 degrees. The prism-cover test demonstrated a deviation angle of −18 prism diopter at the primary gaze, upward 25 degrees, and downward 25 degrees, excluding the possibility of A- or V-pattern exotropia. Additionally, the patient's eye movement function was normal, with no external ocular muscle dysfunction, and no cataracts were observed. He had no history of issues affecting his growth or intellectual development. His parents were non-consanguineous and had no history of hereditary or metabolic diseases. Neither the parents nor the sister exhibited any symptoms.

#### 2.2.2 SEM examination of the hair

The structural characteristics of the patient's hair were examined by SEM, which revealed incomplete or abnormal keratinization of the hair shafts, characterized by irregular, jagged scales and lifted edges (Figure 1C). These structural anomalies indicated compromised cuticle integrity and surface irregularities, which contributed to the overall fragility and decreased density of the hair.

#### 2.2.3 Identification of compound heterozygous variants in the *LSS* gene

**Figure 2:**
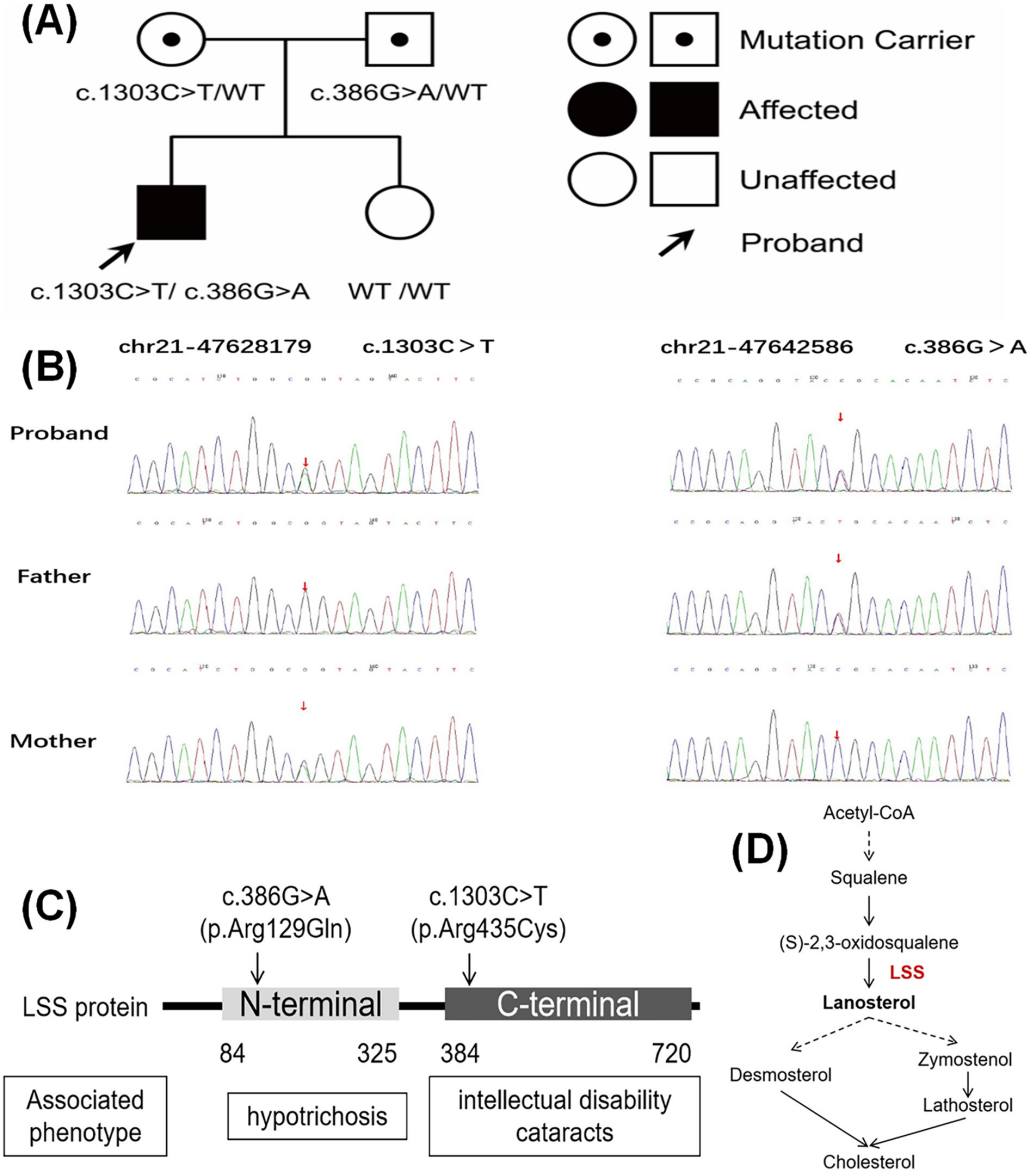
Genetic sequencing results of the patient. **(A)** Pedigree chart. Individuals highlighted in black have clinical symptoms, with the proband indicated by the arrow. **(B)** Sanger sequencing results for the pathogenic variant gene of the proband and his parents. **(C)** Diagram of the detected mutation sites. **(D)** Schematic diagram of LSS-catalyzed cholesterol synthesis.

The following compound heterozygous missense variants in the *LSS* gene were detected and confirmed by Sanger sequencing in the proband: c.1303C>T (p.Arg435Cys) in exon 14 and c.386G>A (p.Arg129Gln) in exon 4 (see Figure 2 and Supplementary Figure 2 for details). The father carried a heterozygous variant c.1303C>T, while the mother carried the heterozygous variant c.386G>A. The sister did not carry either variant. According to the 2015 ACMG guidelines, these missense variants are likely pathogenic (see Supplementary Table 1). The pathogenicity analysis for c.1303C>T is supported by PM2_Supporting (low-frequency variation in normal population databases), PM3_Strong [reported in the literature in cases of recessive inheritance (14)], and PP4 (the patient's phenotype or family history is highly specific for a disease with a single genetic etiology). For c.386G>A, the pathogenicity is supported by PM2_Supporting, PM3_Strong (two cases reported in the MyGenostics database), and PP4. The locations of these two variants within the *LSS* gene and a schematic of the LSS protein's role in catalyzing cholesterol biosynthesis are shown in Figures 2C,D, respectively.

#### 2.2.4 Alteration in LSS protein structure

**Figure 3:**
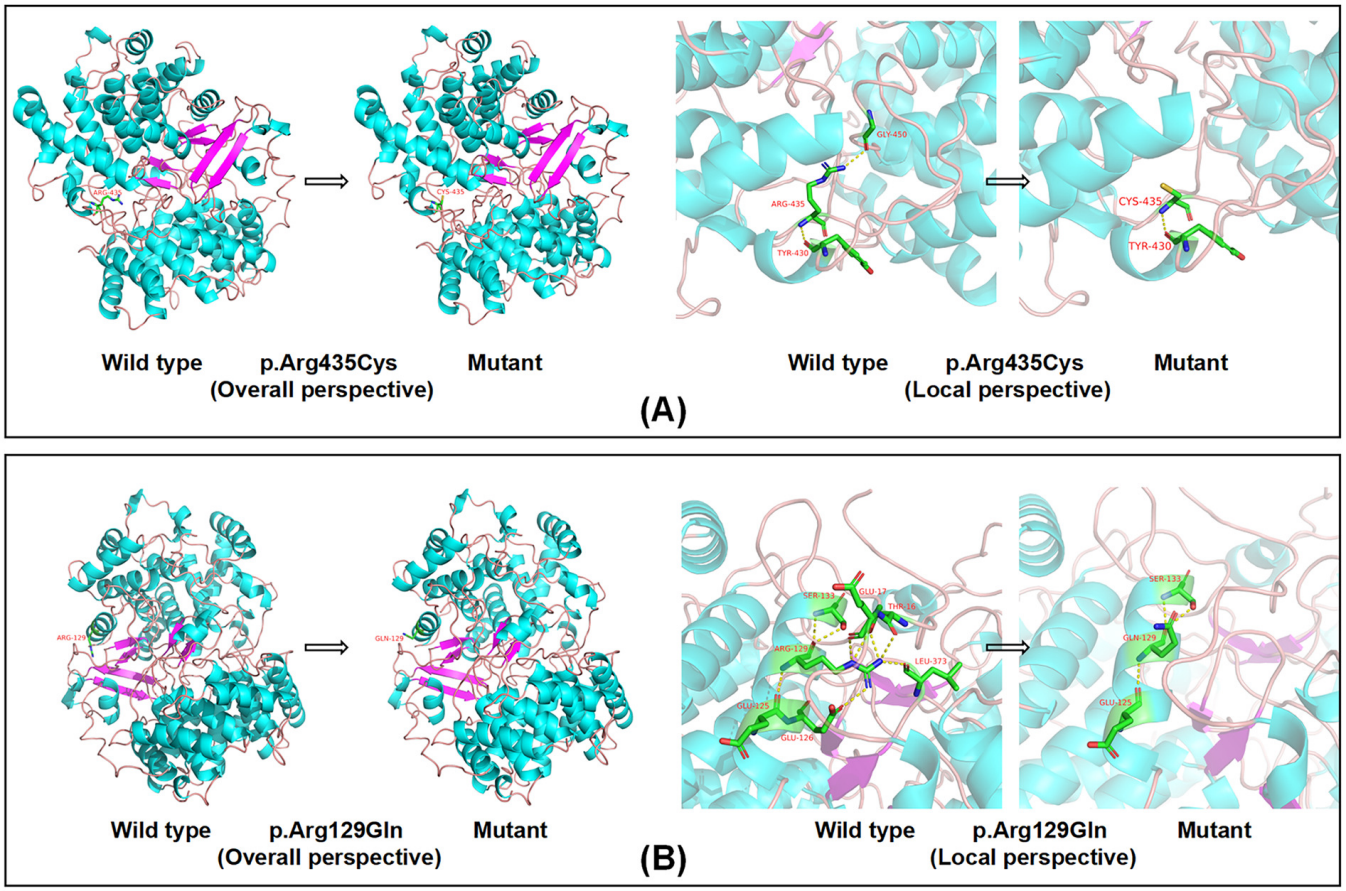
Computational modeling of *LSS* variants. **(A)** The Arg435>Cys substitution in LSS alters the side chain from basic to polar neutral, causing the loss of the hydrogen bond with the amino acid at position 450. **(B)** The Arg129>Gln substitution in LSS alters the amino acid side chain from a basic to a polar neutral residue, resulting in a change in the hydrogen bonding pattern.

Computational modeling was performed to assess the impact of the identified *LSS* variants on synthase integrity. The c.1303C>T (p.Arg435Cys) variant was predicted to result in the substitution of arginine with cysteine, which changes a basic amino acid to a polar neutral one in the side chain and disrupts the hydrogen bond with amino acid 450 (Figure 3A). Similarly, the c.386G>A (p.Arg129Gln) variant was predicted to lead to the substitution of arginine with glutamine, which changes a basic amino acid to a polar neutral one in the side chain and modifies the hydrogen bonding pattern (Figure 3B). The loss of the hydrogen bond at amino acid 450 is expected to affect the stability of the LSS protein. Additionally, these variants may influence the local arrangement of the helix around the lanosterol-binding site, potentially impacting the enzyme's catalytic activity. The results of the patient's conservation analysis and other bioinformatics analyses are provided in Supplementary Figure 3 and Table 2.

#### 2.2.5 Literature review

Relevant cases of *LSS* variants were identified using the keywords “*LSS*” and “lanosterol synthase” in the Human Gene Mutation Database (HGMD, https://www.hgmd.cf.ac.uk/ac/index.php), Orphanet database, PubMed, and Chinese databases, up to August 2024. To date, 63 distinct variants of the *LSS* gene have been reported, comprising 53 missense variants (84.1%), 4 splice variants (6.3%), 3 insertion variants (4.8%), and one each of nonsense (1.6%), synonymous (1.6%), and small deletion variants (1.6%). The most common clinical phenotypes associated with these variants include hypotrichosis (48 cases, 73.0%), intellectual disability (21 cases, 33.3%), cataracts (6 cases, 9.5%), palmoplantar keratoderma (6 cases, 9.5%), autism spectrum disorder (3 cases, 4.8%), epilepsy (2 cases, 3.2%), increased endogenous ouabain concentration (1 case, 1.6%), and white matter disease (1 case, 1.6%). Among these, 29 variants (46.0%) were located in the C-terminal domain (amino acids 384–720), with 16 cases (16/29, 55.2%) presenting with intellectual disability or cataracts. Conversely, 34 variants were located outside the N-terminal domain, with 26 cases (26/34, 76.5%) primarily exhibiting hypotrichosis.

## 3 Discussion

The *LSS* gene, located on chromosome 21q22.3 and consisting of 23 exons, encodes LSS, a key enzyme in cholesterol biosynthesis. This enzyme catalyzes the conversion of (S)-2,3-oxidosqualene to lanosterol, which is crucial for embryonic development and tissue growth (1, 2, 7). Defects in *LSS* function are known to impair cholesterol production and lead to a variety of disorders (1, 2, 9, 11). In the present case, a patient with congenital hypotrichosis also exhibited rare strabismus but no cataracts. Whole-exome sequencing identified compound heterozygous variants in the *LSS* gene, including a novel c.386G>A (p.Arg129Gln) variant. Functional analysis and reference to the ACMG guidelines supported the pathogenicity of these variants. This case broadens the understanding of *LSS* gene variants by expanding the spectrum of associated clinical phenotypes and variants.

*LSS* variants exhibit considerable phenotypic heterogeneity, initially linked to hypotrichosis (OMIM 618275; ORPHA 55654) (2, 6) and later associated with alopecia–intellectual disability syndrome (OMIM 618840; ORPHA 2850), cataracts (OMIM 616509; ORPHA 91492; ORPHA 98994), and palmoplantar keratoderma–congenital alopecia syndrome (OMIM 212360; ORPHA 1366), all of which are inherited as autosomal recessive genetic disorders. Previous immunohistochemical analysis identified LSS protein expression in the outer root sheath and hair matrix of hair follicles, suggesting that *LSS* variants might contribute to hair loss (7). *LSS* variants have also been shown to cause hypotrichosis with or without intellectual disability, congenital cataracts, or cognitive impairment (2, 11, 15). However, the genotype–phenotype correlations remain unclear, particularly regarding which variants lead solely to alopecia and which are associated with more severe neurodevelopmental and dermatological symptoms.

The LSS protein consists of 732 amino acids and features two major domains: the N-terminal domain (amino acids 84–325) and the C-terminal domain (amino acids 384–720) (7). Variants in the N-terminal region of the *LSS* gene are thought to be associated with hair loss, whereas those in the C-terminal region are more commonly linked to ocular abnormalities. Among the 63 variants reported in HGMD, 46% were located within the C-terminal domain, with 55.2% of these cases presenting with intellectual disability or cataracts. In contrast, variants outside the N-terminal domain were more commonly associated with hypotrichosis (76.5%). While strabismus is typically multifactorial, involving genetic, environmental, and physiological factors, its specific association with *LSS* gene variants remains underexplored. In the present case, we identified compound heterozygous variants, c.1303C>T (p.Arg435Cys) and c.386G>A (p.Arg129Gln), in the C- and N-terminal regions, respectively. These findings partially explain the presence of hypotrichosis in our patient, and further research is needed to explore the potential connection between *LSS* gene variants and ocular developmental abnormalities.

Protein structure modeling, such as using the SWISS-MODEL platform, provides significant insights into the structural and functional consequences of variants. For our patient, modeling predicted that the identified variants cause substantial alterations in hydrogen bonding, potentially disrupting the three-dimensional structure of the LSS protein. These structural changes may impair the enzymatic activity of LSS, affecting its role in cholesterol biosynthesis.

This report describes a rare case of congenital hypotrichosis with associated strabismus in a Chinese patient and introduces a novel *LSS* variant, c.386G>A (p.Arg129Gln). The presented case underscores the clinical heterogeneity of *LSS*-related disorders. Additionally, the findings of SEM analysis indicate that the c.1303C>T (p.Arg435Cys) and c.386G>A (p.Arg129Gln) variants cause hair shaft abnormalities. Further research is needed to elucidate the mechanisms and functions of these variants, as well as their impact on cholesterol metabolism and hair growth. Investigation of the allelic series at these loci will enhance our understanding of the genotype-phenotype relationship in disorders linked to the *LSS* gene.

## Data Availability

The original contributions presented in the study are included in the article/Supplementary Material, further inquiries can be directed to the corresponding authors.
