# Supplementary material for: Congenital hypotrichosis caused by compound heterozygous variants in the LSS gene in a Chinese patient with strabismus: case report
Source: Front Pediatr. 2025 Apr 29;13:1512646. doi: 10.3389/fped.2025.1512646 (PMC12069456; doi:10.3389/fped.2025.1512646)
Supplement: Supplementary file 1 [file Supplementaryfile1.docx]

**Supplementary Figure 1.** The eye position chart of the patient showing eye movements in all directions.

Note: Eye movements: Both eyes exhibited coordinated movements in all directions, with no obvious restriction or overactivity of the extraocular muscles.


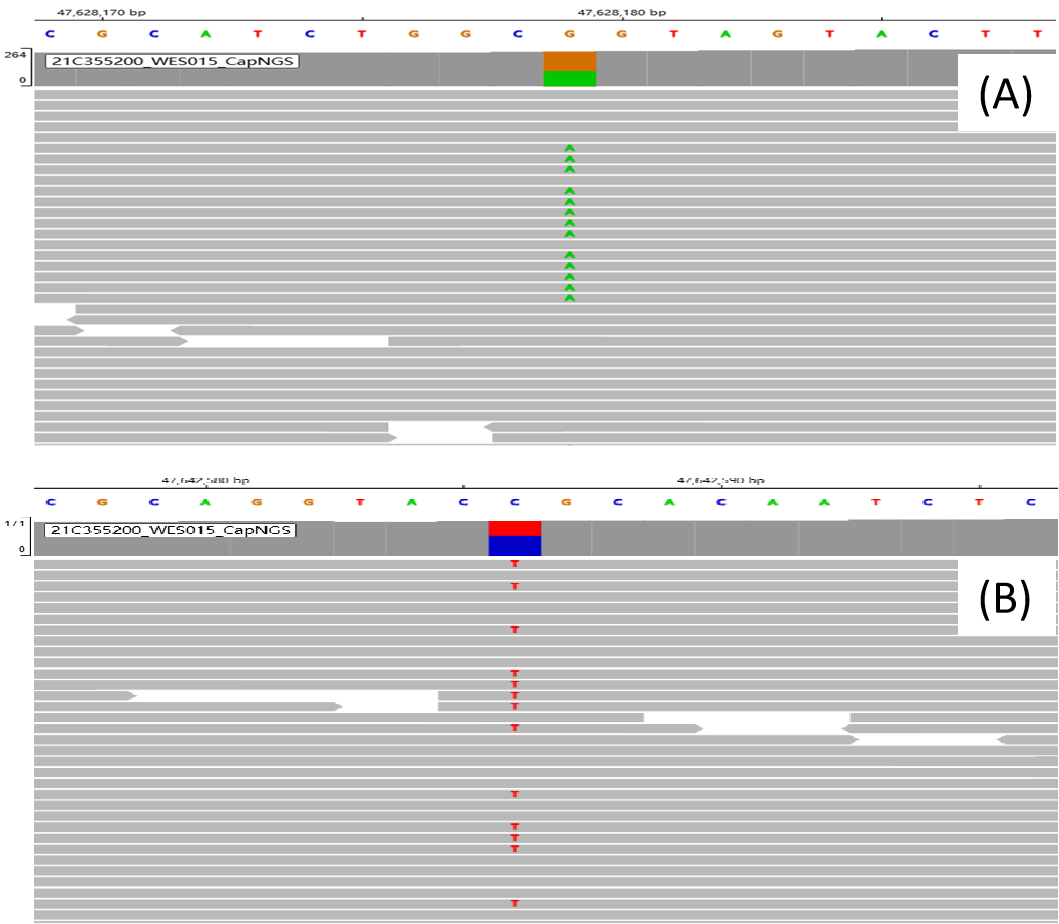


**Supplementary Figure 2.** Integrative Genomics Viewer (IGV) results of the LSS gene mutation sites in the patient.

Note: (A) c.1303C>T, p.Arg435Cys; (B) c.386G>A, p.Arg129Gln.


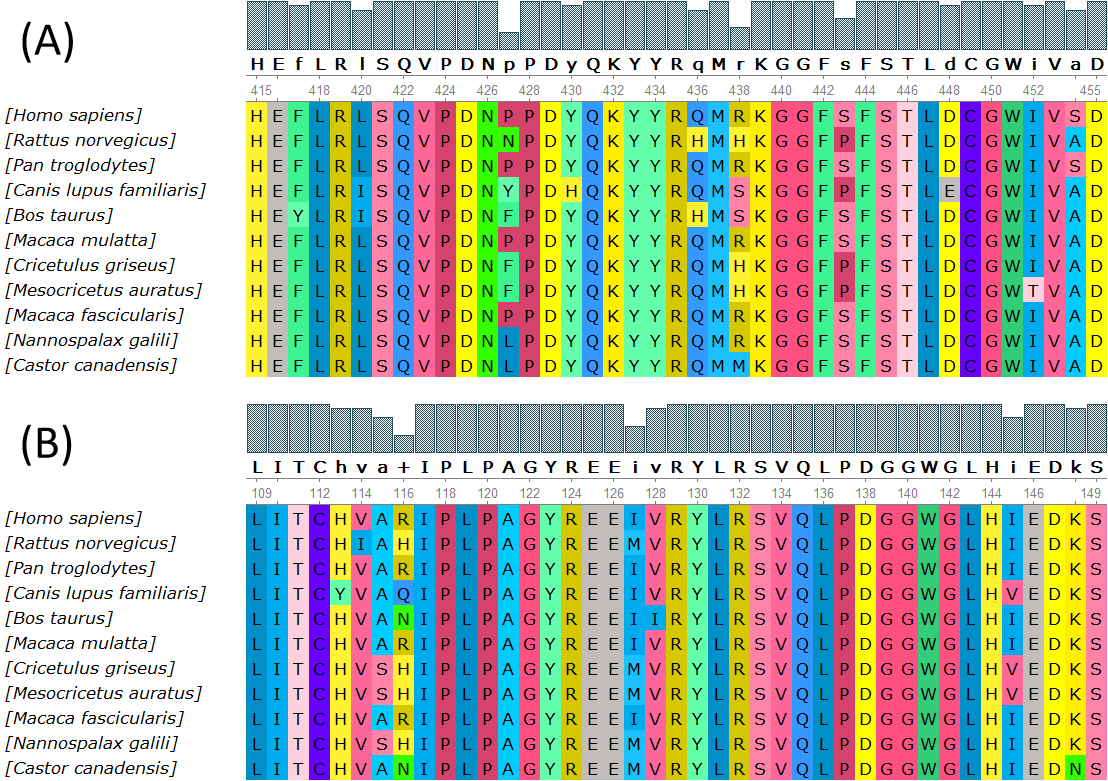


**Supplementary Figure 3.** Conserved sequence analysis of LSS gene variant sites in the patient.

Note: (A) c.1303C>T, p.Arg435Cys; (B) c.386G>A, p.Arg129Gln.

**Supplementary Table 1.** Information on the LSS gene variants in this patient

| **Location** | **Exon** | **Nucleotide/**  **Amino acid** | **Homozygous/Heterozygous** | **Normal Frequency** | **ACMG pathogenicity** | **Evidence** | **Origin** |
| --- | --- | --- | --- | --- | --- | --- | --- |
| chr21:476  28179 | 14 | c.1303C>T  (p.Arg435Cys) | Heterozygous | 0.000788 | Likely  pathogenic | PM2_Supporting+PM3_Strong+PP4 | Father |
| chr21:476  42586 | 4 | c.386G>A  (p.Arg129Gln) | Heterozygous | 0.0002175 | Likely  pathogenic | PM2_Supporting+PM3_Strong+PP4 | Mother |

**Supplementary Table 2.** Software prediction results of the pathogenicity of LSS variant sites in the patient

| **Tools** | **c.1303C>T** | **c.386G>A** |
| --- | --- | --- |
| REVEL | U (0.433) | U (0.531) |
| Polyphen2 | Probably_damaging (0.995) | Probably_damaging (1) |
| MutationTaster | Disease_causing (1) | Disease_causing (1) |
| MCAP | P (0.08029362) | P (0.05610480) |
| SIFT | Damaging (0.001) | Damaging (0.023) |
| LRT | D (0) | D (0) |
| GERP | Conserved (4.6) | Conserved (5.07) |
| SPIDEX | -3.5729 | -1.2318 |
| ClinPred | 0.98457772 | 0.98515630 |
| AlphaMissense | ambiguous (0.4649) | ambiguous (0.5368) |
